# Clarifying directional dependence among measures of early auditory processing and cognition in schizophrenia: leveraging Gaussian graphical models and Bayesian networks

**DOI:** 10.1017/S0033291724000023

**Published:** 2024-07

**Authors:** Samuel J. Abplanalp, David L. Braff, Gregory A. Light, Yash B. Joshi, Keith H. Nuechterlein, Michael F. Green

**Affiliations:** 1 Desert Pacific Mental Illness Research, Education and Clinical Center, Veterans Affairs Greater Los Angeles Healthcare System, Los Angeles, CA, USA; 2 Department of Psychiatry and Biobehavioral Sciences, University of California Los Angeles, Los Angeles, CA, USA; 3 Desert Pacific Mental Illness Research Education and Clinical Center, VA San Diego Healthcare System, San Diego, CA, USA; 4 Department of Psychiatry, University of California, San Diego, La Jolla, CA, USA

**Keywords:** Bayesian network, cognition, directed acyclic graph, early auditory processing, mismatch negativity, schizophrenia

## Abstract

**Background:**

Research using latent variable models demonstrates that pre-attentive measures of early auditory processing (EAP) and cognition may initiate a cascading effect on daily functioning in schizophrenia. However, such models fail to account for relationships among individual measures of cognition and EAP, thereby limiting their utility. Hence, EAP and cognition may function as complementary and interacting measures of brain function rather than independent stages of information processing. Here, we apply a data-driven approach to identifying directional relationships among neurophysiologic and cognitive variables.

**Methods:**

Using data from the Consortium on the Genetics of Schizophrenia 2, we estimated Gaussian Graphical Models and Bayesian networks to examine undirected and directed connections between measures of EAP, including mismatch negativity and P3a, and cognition in 663 outpatients with schizophrenia and 630 control participants.

**Results:**

Chain structures emerged among EAP and attention/vigilance measures in schizophrenia and control groups. Concerning differences between the groups, object memory was an influential variable in schizophrenia upon which other cognitive domains depended, and working memory was an influential variable in controls.

**Conclusions:**

Measures of EAP and attention/vigilance are conditionally independent of other cognitive domains that were used in this study. Findings also revealed additional causal assumptions among measures of cognition that could help guide statistical control and ultimately help identify early-stage targets or surrogate endpoints in schizophrenia.

## Introduction

Cognitive impairments in schizophrenia are associated with deficits in early perceptual processing, including early auditory processing (EAP). Studies have demonstrated that EAP impacts cognition and daily functioning in schizophrenia (Green, Hellemann, Horan, Lee, & Wynn, 2012; Javitt, 2009; Koshiyama et al., 2020; Rassovsky, Horan, Lee, Sergi, & Green, 2011; Thomas et al., 2017). For instance, a large-sample structural equation model (SEM) from the Consortium on the Genetics of Schizophrenia 2 (COGS-2) showed that EAP deficits are related to cognitive impairments and indirectly associated with negative symptoms and reduced functional outcomes (Thomas et al., 2017). Hence, EAP and cognition could be pivotal in initiating a cascading effect on daily functioning. Clarifying the associations between EAP and cognition could help guide proper statistical control of these variables and help identify early-stage intervention targets or surrogate endpoints in schizophrenia.

Previous studies have predominantly used latent variable models, such as SEM, to examine the association between early perception and cognition (Green et al., 2012; Koshiyama et al., 2020; Rassovsky et al., 2011; Thomas et al., 2017); however, these analyses require challenging assumptions. Latent variable models assume (1) a common cause underlies the observed variables and (2) *local independence*, such that observed indicators must be independent after accounting for their latent variables (Bollen & Bauldry, 2011; Borsboom, Mellenbergh, & van Heerden, 2003; Rhemtulla, van Bork, & Borsboom, 2020). Yet, research on the interplay of EAP and cognitive domains suggests interdependency.

Mismatch negativity (MMN) and P3a are among the most studied event-related brain potentials in schizophrenia (Erickson, Ruffle, & Gold, 2016; Koshiyama et al., 2018; Light et al., 2015; Light & Braff, 2005; Light & Näätänen, 2013; Wynn, Sugar, Horan, Kern, & Green, 2010). Although earlier components of EAP exist (e.g. prepulse inhibition), MMN and P3a reliably index pre-attentive processing and perception. However, because MMN and P3a are sequentially evoked as a response complex, they are not entirely independent (Braff & Light, 2004; Giordano et al., 2021; Leitman et al., 2010). For example, a path analysis demonstrated that a schizophrenia diagnosis is associated with deficits in MMN, resulting in reduced P3a (Leitman et al., 2010). Notably, the model was not significant when the order of MMN and P3a was reversed. Beyond EAP, the interdependence among cognitive domains is well recognized. For instance, Digit Symbol tasks, often conceptualized as primarily measuring processing speed, require additional cognitive processes for successful task performance, including working memory and visual attention shifts (Abplanalp et al., 2023; Gold, Hahn, Strauss, & Waltz, 2009; Sheffield & Barch, 2016). Impairments in any of these processes could result in poor task performance.

The potential interdependence of EAP and cognition suggests that these constructs may be better represented as read-outs of interacting brain systems under diverse levels of cognitive challenges (i.e. passive *v.* active cognitive tasks) rather than as latent variables. Two methods are well-suited to analyze such interdependencies. The first is Gaussian Graphical Models (GGMs). GGMs represent constructs as interacting nodes, in which nodes symbolize variables, and the edges between nodes denote the strength of association between variables after accounting for all other variables in the network (Abplanalp & Green, 2022; Borsboom et al., 2021; Epskamp, Borsboom, & Fried, 2018a; Epskamp, Waldorp, Mõttus, & Borsboom, 2018b). However, GGMs have a limitation – they are *undirected*. That is, GGMs do not estimate the direction of association (Briganti, Scutari, & McNally, 2022).

A complementary method to GGMs is Bayesian network analysis – a *directed* network. Bayesian networks represent nodes using Directed Acyclic Graphs (DAGs), mathematical objects that indicate the conditional probability among nodes (Briganti et al., 2022; Geiger, Verma, & Pearl, 1990; Pearl, 1998; Pearl & Mackenzie, 2019; Verma & Pearl, 2022). For continuous variables, Bayesian networks estimate *arcs* using Gaussian distributions, allowing the distributions to be modeled as linear regressions (Briganti et al., 2022). The influence of one node, called ‘parents,’ on another node, called ‘descendants’, is estimated by a unit change in the parent node's regression coefficient. Conversely, if two nodes are unconnected, they are *conditionally independent*. Cross-sectional DAGs can illustrate various dependent structures among nodes and provide insights beyond GGMs (Grosz, Rohrer, & Thoemmes, 2020; Rohrer, 2018; Wysocki, Lawson, & Rhemtulla, 2022).

An arrow between two variables in a Bayesian network signifies more than correlation. Specifically, an arrow represents a conditional dependency with direction. If there is an arc from A to B, it suggests that A directly influences B even when all other variables in the network are accounted for. Correlation can flow in any direction, but causation only flows in the direction of the arrows (Pearl & Mackenzie, 2019). Following DAG terminology, we can infer three types of dependent structures. The first structure is a chain (A → B → C), where A has an indirect influence on C through B, making A and C conditionally independent when conditioning on B (A ⊥ C | B). The second structure is a confounder (A ← C → B), where C is a common cause of A and B, making A and B conditionally independent when controlling for C (A ⊥ B | C). The third structure is a collider (A → C ← B), with no association between A and B (A ⊥ B). Collider structures are critical because spurious associations may emerge between A and B if C is not accounted for.

This study used data from COGS-2 (Swerdlow, Gur, & Braff, 2015) to examine the interdependence between EAP and cognition in schizophrenia patients and controls. We selected variables based on those used in the previous SEM paper on the same dataset (Thomas et al., 2017) to highlight how associations between EAP and cognition differ when using latent variable models *v.* GGMs and Bayesian networks. Our analyses had two primary goals. First, we used GGMs to assess the network structure of EAP and cognition and to determine whether the structure differed between schizophrenia patients and controls. Second, we applied Bayesian network analyses to clarify the direction of network connections. We did this separately by group to identify parent variables and evaluate differences in dependent structures.

## Methods

### Participants

The study included 1415 patients diagnosed with schizophrenia or schizoaffective disorder, depressed type, and 1062 healthy community controls. We used the Structured Clinical Interview for DSM-IV (First & Gibbon, 2004) to confirm diagnoses. Participants were recruited across five sites: the University of California, Los Angeles; University of California, San Diego; Mount Sinai School of Medicine, New York; University of Pennsylvania; and University of Washington, Seattle. Exclusion criteria included: neurologic or additional Axis I psychiatric disorders; head injury; stroke; and substance abuse. Each site's local Institutional Review Boards approved the study, and all participants provided written informed consent. Details regarding recruitment, participant selection criteria, and clinical assessments are presented elsewhere (Abplanalp, Braff, Light, Nuechterlein, & Green, 2022; Greenwood et al., 2019; Joshi et al., 2023; Lee et al., 2020; Swerdlow et al., 2015).

### Mismatch negativity and P3a

In this study, participants experienced binaural tones by inserting earphones. The tones were set at 1 kHz and 85 dB, featured a 1 ms rise and fall time, and were set to occur every 500 ms. We employed a duration-deviant auditory oddball paradigm, wherein the deviant stimuli's duration differed. A pseudorandom sequence was used to present standard tones, which had a 90% probability and a duration of 50 ms, and deviant tones, which had a 10% probability and lasted 100 ms. A minimum of six standard stimuli was ensured before introducing each deviant stimulus. To determine the MMN/P3a waveform, we subtracted the ERP waveform resulting from standard stimuli from the ERP waveform induced by deviant stimuli. The MMN and P3a amplitudes were measured as the average amplitude within the 135–205 ms and 250–300 ms time windows, respectively (Light et al., 2015).

### Cognition

Selected measures of cognition were taken from the prior COGS-2 SEM paper and are defined in Table 1. We measured attention/vigilance with the Degraded Stimulus (DS-CPT) and Identical Pairs (CPT-IP) Continuous Performance Tests (Green & Swets, 1966; Nuechterlein et al., 2015); face and object memory with the Penn Face Memory task (PFMT) and the Visual Object Learning Test (VOLT) from the Penn Computerized Neurocognitive Battery (Gur et al., 2001, 2010, 2015; Moore, Reise, Gur, Hakonarson, & Gur, 2015); verbal learning and recognition with the California Verbal Learning Test-Second Edition (CVLT) (Stone et al., 2015; Woods, Delis, Scott, Kramer, & Holdnack, 2006) and the Penn Word Memory task (PWMT) from the Penn Computerized Neurocognitive Battery (Gur et al., 2001, 2010, 2015; Moore et al., 2015); and working memory with the Letter N-back task (N-back) from the Penn Computerized Neurocognitive Battery (Gur et al., 2001, 2010, 2015; Moore et al., 2015) and the Letter-Number Span Task-Forward (LNS-F) and Letter-Number Span Task-Reorder (LNS-R) from the Wechsler Memory Scale-Third Edition (Gold, Carpenter, Randolph, Goldberg, & Weinberger, 1997; Lee et al., 2015; Wechsler, 1997).

**Table 1.** Names and descriptions of the cognitive tasks

Cognitive domain	Task bame	Description
Attention/vigilance	Continuous performance test (degraded stimulus)	Participants view a series of blurred single digits that were presented for 29-ms each at a rate of one digit per second and asked to detect each target ‘0’. Dependent variable = *d*’, a signal/noise discrimination index
Attention/vigilance	Continuous performance test (identical pairs)	Participants view a series of digits for 50 ms in a quasi-random sequence at a rate of one digit per second and are asked to respond when two digits appear twice in a row. Dependent variable = *d*’, a signal/noise discrimination index.
Face and spatial memory	Penn Face Memory task	Participants view 20 faces one at a time and are asked to memorize them. During the recognition phase, participants are shown 40 faces, 20 of which were the faces they were shown, and the other 20 were distractors. Dependent variable = face accuracy.
Object memory	Visual object learning test	Same as Penn Face Memory task, but instead of faces participants memorize shapes.
Verbal learning and recognition	California verbal learning test	Participants recall as many words as possible from a list of 16 words across five trials. Dependent variable = total number correct.
Verbal learning and recognition	Penn word Memory task	Same as Penn Face Memory task, but instead of faces participants memorize words.
Working memory	Letter N-back task	Participants attend to a continual series of letters that flash on the screen and press the spacebar according to three different rules (0-back, 1-back, and 2-back). Dependent variable = total accuracy.
Working memory	Letter-number span task forward	Participants repeat a series of letters and numbers in the same order as they were presented. Dependent variable = total number correct.
Working memory	Letter-number span task reorder	Participants repeat a series of digits in ascending order and letters in alphabetical order. Dependent variable = total number correct.

### Statistical Analyses

#### Gaussian graphical models (undirected networks)

All analyses were performed using R (Version 4.2.0), with code available at: https://osf.io/h78jv/. We used *bootnet* (Epskamp et al., 2018a, 2018b) to estimate GGMs. Nodes comprised EAP and cognitive variables and lines between nodes are edges, representing undirected (A ─ B) partial correlations. All variables were standardized using *z*-score transformations. The primary distinction between a standard partial correlation and the ones estimated by GGMs is the use of a regularization technique. We applied regularization using the Extended Bayesian Information Criterion form of the graphical least absolute shrinkage with a tuning parameter gamma of 0.5 (Epskamp et al., 2018a, 2018b). This method employs an L1 penalty that estimates a sparse inverse covariance matrix and shrinks trivially small partial correlations to zero, omitting them from the graph. Hence, there is a higher degree of confidence in the estimated edges within GGMs compared to standard partial correlations. To illustrate how associations among measures of EAP and cognition can differ among correlation types, we also estimated bivariate correlation and standard partial correlation (without regularization) networks (see online Supplementary Material).

We then evaluated accuracy of the GGM edges by using nonparametric bootstrapping with 1000 bootstrap samples. Lastly, we measured node predictability (Haslbeck & Fried, 2017). Node predictability represents the shared variance of each node and is an absolute measure of interconnectedness. Predictability is illustrated in black around each node. GGMs were arranged in a circular layout using the R package *qgraph* (Epskamp, Cramer, Waldorp, Schmittmann, & Borsboom, 2012).

#### GGM network comparison

To assess whether the GGMs between schizophrenia patients and controls significantly differed, we conducted network comparison tests with 2000 iterations via the *NetworkComparisionTest* (van Borkulo et al., 2022). We used the *Network Invariance* Test to compare the groups' overall network structure. This test evaluates if there are differences in network edges between groups, and whether those differences are significant. Edges may appear to differ between groups but that does not automatically indicate that the differences are statistically significant. We used the *Global Strength Invariance* Test to compare the sum of all absolute edge values. Both invariance tests were conducted using the Holm-Bonferroni method for multiple comparisons.

#### Bayesian networks (directed networks)

Bayesian networks include variables represented as nodes via DAGs and are connected via arcs. An arc from one node to another implies a directed connection – which could be positive or negative. However, Bayesian networks rely on critical assumptions, including the presence of no bidirectional causal relationships (A → B and B → A) or feedback loops (A → B, B → C, and C → A), and that all essential variables are included in the network (Geiger et al., 1990; McNally, Robinaugh, Deckersbach, Sylvia, & Nierenberg, 2022; Pearl, 1998; Verma & Pearl, 2022). The assumption of all essential variables is quite difficult to satisfy, given the number of third variables that could affect cognition. Hence, we estimated DAGs using the same variables as the GGMs. We again standardized all variables.

We used a score-based learning algorithm called the *hill-climbing* (Russell, 2010) algorithm in *bnlearn* (Scutari, 2010) to estimate Bayesian networks. The hill-climbing algorithm is a machine-learning process that estimates DAGs by exploring single-edge additions, removals, and reversals to optimize goodness of fit based on the Bayesian Information Criteria (BIC). First, it calculates the fit of an empty DAG, defined as Score (*G*), and sets maxscore = Score(*G*). Next, it computes the modified network G* score for edge additions, deletions, or reversals that do not result in a cyclic network. If any G* has a Score (G*) > Score(*G*), maxscore is updated to Score (G*), and G* becomes the new candidate network; this process repeats until an optimal fitting DAG is selected.

To ensure network stability, we used a bootstrapping method with 1000 samples (Abplanalp et al., 2023; McNally, Heeren, & Robinaugh, 2017a; McNally, Mair, Mugno, & Riemann, 2017b). The technique involved creating a network for each sample, averaging all networks, and examining the frequency and direction of arcs. If an arc was present (regardless of direction) in a minimum of 85% of the bootstrapped DAGs and pointed in a given direction in at least 51% of those DAGs, it was represented in the final Bayesian network. We also computed the standardized Beta coefficient for each arc of the final network, indicating the degree of influence a unit change in a parent node has on descendant nodes, and the overall fit of Bayesian networks via the BIC. The Bayesian networks were arranged in the same circular layout as the GGM to facilitate comparison.

#### Bayesian network comparison

We used a form of the Jaccard similarity coefficient to examine differences in Bayesian network structure. This metric divides the number of arcs that are common in both networks by the number of unique arcs across both networks. We also computed a metric called Arc direction agreement to examine differences in arc direction. This metric divides the number of arcs that have the same direction in both networks by the total number of arcs in one of the networks. The Jaccard similarity coefficient and Arc direction agreement range between 0 and 1. Both metrics were bootstrapped using 1000 iterations.

## Results

After excluding participants via listwise deletion, the sample included 663 outpatients diagnosed with schizophrenia or schizoaffective disorder and 630 community controls. Missing data were primarily due to the EAP measures being added later in the COGS-2 study. Demographic information is presented in Table 2. Compared to controls, schizophrenia patients were significantly older, *t*(1291) = −10.37, *p* < 0.001, had a higher proportion of males χ^2^(1, *N* = 1293) = 55.80, *p* < 0.001, and had a lower proportion of Asian and White participants and a higher proportion of Black participants, χ^2^(6, *N* = 1293) = 77.34, *p* < 0.001.

**Table 2.** Demographic characteristic of schizophrenia patients and controls

	Schizophrenia (*n* = 663)	Controls (*n* = 630)
Age* (mean [s.d.])	46.30 (11.38)	39.29 (12.91)
Age of illness onset (mean [s.d.])	22.64 (7.45)	N/A
Education* (mean [s.d.])	12.77 (2.04)	14.89 (2.24)
Race (*%*)		
Asian*	3.47	8.57
Black*	37.71	20.16
Did not identify	0.0	0.32
More than one race	14.18	11.59
Native American	1.20	0.79
Pacific Islander	1.06	0.32
White*	42.38	58.25
Sex* (*%*)		
Female	28.81	49.05
Male	71.19	50.95

*Note*. * = Significantly different at *p* < 0.001.

### Gaussian graphical models

In schizophrenia, the strongest edges were between LNS-F and LNS-R (*r*_p_ = 0.49) and mismatch negativity and P3a (*r*_p_ = −0.54). LNS-F and LNS-R (*r*_p_ = 0.50) and mismatch negativity and P3a (*r*_p_ = −0.48) were also the strongest edges in controls. The confidence intervals were narrow in both groups for the nonparametric bootstrap analysis, indicating stable estimates (see online Supplementary Material).

**Figure 1.**
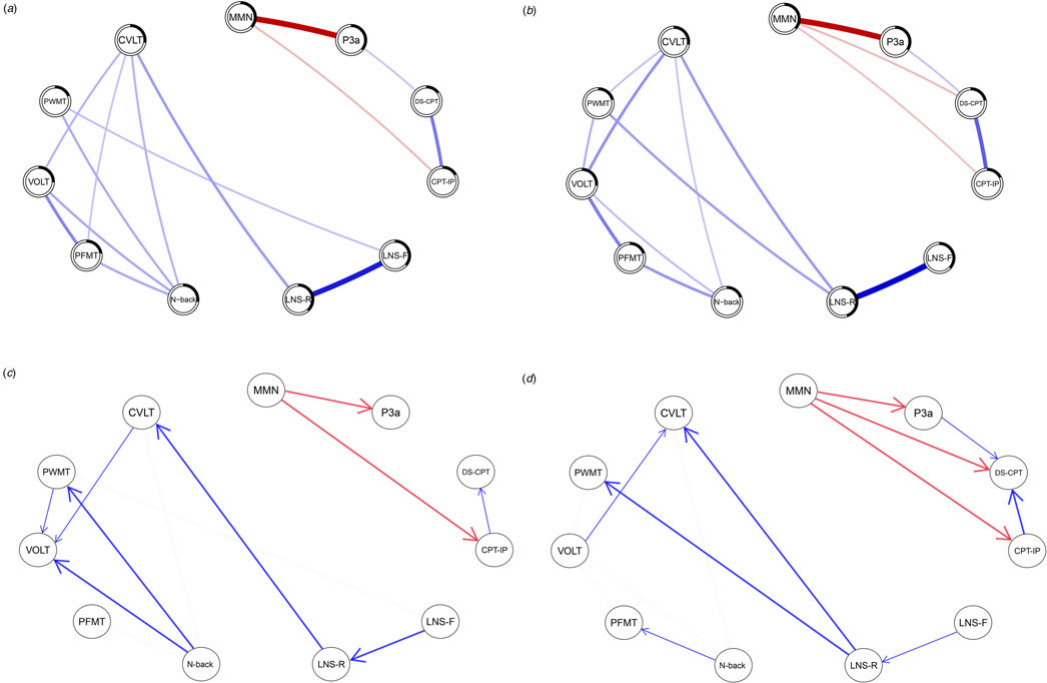
GGMs and Bayesian networks of EAP and cognitive variables for Schizophrenia patients and controls. *Note*. Panel A, GGM for controls; Panel B, GGM for schizophrenia patients; Panel C, Bayesian network for controls; Panel D, Bayesian network for schizophrenia patients. GGM, Gaussian graphical model; EAP, early auditory processing; MMN, mismatch negativity; DS-CPT, degraded stimulus continuous performance test; CPT-IP, continuous performance test identical pairs; LNS-F, letter-number span task forward; LNS-R, letter-number span task reorder; PWMT, Penn Word Memory task; CVLT, California Verbal Learning Test; N-back, Letter N-back task; PFMT, Penn Face Memory task; VOLT, Visual Object Learning Test.

In both groups, EAP and attention/vigilance variables were connected but separated from other cognitive domains (Fig. 1). The CVLT and the VOLT had the most connections in schizophrenia patients, and the CVLT and the N-back had the most connections to other variables in controls. The LNS-R had the highest node predictability in both groups.

### GGM network comparison test

The Global Network Invariance Test indicated no significant differences in the structure between the schizophrenia and control network, even though six edges were different between the baseline networks (*M* = 0.19, *p* = 0.33). The Global Strength Invariance Test was also non-significant (*S* = 0.18, *p* = 0.53).

### Bayesian networks

Figure 1 also depicts the Bayesian networks for schizophrenia patients and controls. Arc thickness indicates the confidence in the directionality from one node to another, with less thick arcs equating to less confident estimates (Table 3). Blue arcs represent positive Beta coefficients, and red arcs represent negative coefficients. The BIC was −9744.99 for the schizophrenia network and −9290.78 for the control network.

**Table 3.** Arc frequency, arc direction, and beta coefficients for the Bayesian networks

From	To	Arc frequency	Arc direction	Beta coefficient
Schizophrenia				
MMN	P3a	1.00	0.74	−0.60
MMN	DS-CPT	0.95	0.77	−0.13
MMN	CPT-IP	0.91	0.76	−0.17
P3a	DS-CPT	0.86	0.60	0.16
CPT-IP	DS-CPT	1.00	0.66	0.30
LNS-F	LNS-R	1.00	0.60	0.58
LNS-R	PWMT	0.99	0.79	0.26
LNS-R	CVLT	1.00	0.76	0.27
CVLT	N-back	0.85	0.51	0.22
N-back	PFMT	1.00	0.61	0.24
VOLT	PFMT	1.00	0.59	0.32
VOLT	PWMT	0.82	0.72	0.20
VOLT	CVLT	1.00	0.61	0.21
VOLT	N-back	0.91	0.54	0.38
Controls				
MMN	P3a	1.00	0.64	−0.60
MMN	CPT-IP	0.97	0.60	−0.23
CPT-IP	DS-CPT	1.00	0.59	0.34
LNS-F	LNS-R	1.00	0.65	0.58
LNS-F	PWMT	0.89	0.57	0.25
LNS-R	CVLT	1.00	0.71	0.30
N-back	PFMT	0.99	0.55	0.28
N-back	VOLT	0.99	0.64	0.21
N-back	PWMT	0.99	0.63	0.28
N-back	CVLT	0.98	0.52	0.30
PFMT	VOLT	1.00	0.57	0.27
CVLT	VOLT	0.97	0.58	0.19

*Note*. MMN, Mismatch negativity; DS-CPT, Degraded stimulus continuous performance test; CPT-IP, Continuous performance test identical pairs; LNS-F, Letter-number span task forward; LNS-R, Letter-number span task reorder; PWMT, Penn Word Memory task; CVLT, California Verbal Learning Test; N-back, Letter N-back task; PFMT, Penn Face Memory task; VOLT, Visual Object Learning Test; arc frequency refers to the proportion of times that an arc appeared in the bootstrap samples, regardless of direction. Arc direction refers to the proportion of times that the arc pointed in that given direction.

Like the GGMs, EAP and attention/vigilance variables were conditionally independent from the other cognitive domains (EAP ⊥ cognition | attention/vigilance) and conditionally dependent on each other. This means that once controlling for the other cognitive domains, EAP is only associated with attention/vigilance, and attention/vigilance is only associated with EAP. However, EAP is not completely independent from the other cognitive domains given the presence of bivariate associations. The Bayesian networks provide additional information on the direction of these relationships. MMN is a parent variable upon which P3a and the CPT depend. Specifically, MMN was a common cause of P3a, CPT-IP, and DS-CPT in schizophrenia patients. P3a was also a parent variable to DS-CPT in schizophrenia but not in controls. Beta coefficients are included in Table 3.

Regarding the remaining cognitive domains, the groups had differences in parent and descendant variables. In schizophrenia, the VOLT was a parent variable to the CVLT, PFMT, N-Back, and PWMT. In controls, the VOLT was a descendant of the CVLT, PFMT, and N-back. In schizophrenia, the N-back was a parent variable to the PFMT and a descendant variable of the VOLT and CVLT. In controls, the N-back was a parent variable upon which the PFMT, VOLT, PWMT, and CVLT depended.

From these connections, two chains emerged in the control group. One initiates from LNS-F and ends at VOLT (LNS-F → LNS-R → CVLT → VOLT) and the second chain begins from N-back and ends at VOLT (N-back → PFMT → VOLT). These patterns suggest that VOLT is a collider variable with different variables leading to it (e.g. PFMT → VOLT ← CVLT) and functions as a common effect. In contrast, the N-back is a confounding variable (e.g. VOLT ← N-back → PWMT) and is a common cause for more than one variable.

In schizophrenia, two chains also emerged, with one initiating from LNS-F and ending at N-back (LNS-F → LNS-R → CVLT → N-back) and the second initiating from VOLT and ending at N-back (VOLT → CVLT → N-back). In contrast to controls where it functioned as a confounding variable, N-back is a collider in patients (e.g. VOLT → N-back ← CVLT), functioning as a common effect. In contrast to controls where VOLT was a collider, it is a confounder in patients (e.g. PWMT ← VOLT → CVLT) and serves as a common cause.

### Bayesian network comparison

The Jaccard similarity coefficient was 0.23, meaning there is a moderate degree of similarity between the arc sets of the two networks. A score of 0.23 suggests that about 23% of the arcs are common between schizophrenia and control networks. Arc direction agreement was 0.46, indicating that 46% of the common arcs between schizophrenia patients and controls have the same direction. This value suggests that when the same relationships (arcs) are present in both networks, the direction of influence differs nearly half of the time.

### Follow-up analyses

**Figure 2.**
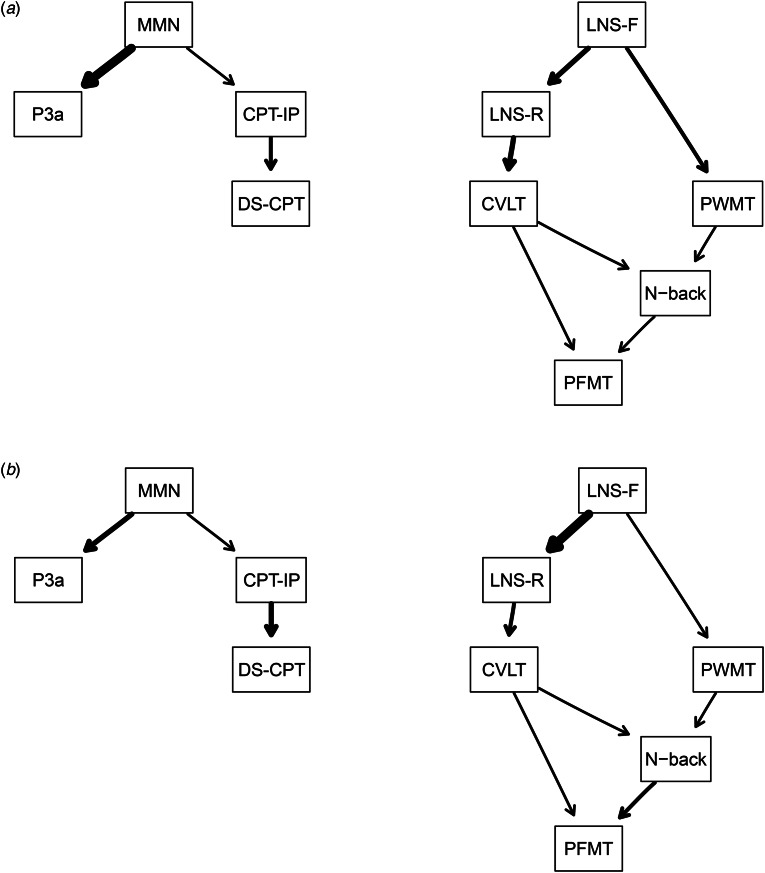
Bayesian networks of EAP and cognitive variables for schizophrenia patients and controls after removing the VOLT. *Note*. Panel A, Bayesian network for controls; Panel B, Bayesian network for schizophrenia patients. EAP, Early auditory processing; VOLT, Visual object learning test; MMN, Mismatch negativity; DS-CPT, Degraded stimulus continuous performance test; CPT-IP, Continuous performance test identical pairs; LNS-F, Letter-number span task Forward; LNS-R, Letter-number span task reorder; PWMT, Penn Word Memory task; CVLT, California Verbal Learning Test; N-back, Letter N-back task; PFMT, Penn Face Memory task.

Based on the Bayesian network results, we conducted follow-up analyses. Given the influential role of the VOLT as a parent variable in schizophrenia and a descendant variable in controls, we estimated Bayesian networks (using the same methods as above) that omitted this domain. After removing the VOLT, the network structures appeared similar in both groups (Fig. 2 in the standard *bnlearn* output). In addition, the VOLT had the strongest between-group effect size of any task used in the study (*d* = 1.32), suggesting that schizophrenia patients were severely impaired in object learning. Moreover, as cognitive function declines with age (Lee et al., 2020), we stratified age to adjust for its confounding effects in the Bayesian networks. These Bayesian networks showed minimal differences from the original networks (online Supplementary Material). Lastly, we conducted regression analyses to illustrate select dependent structures estimated from the Bayesian networks (online Supplementary Material).

## Discussion

This study used GGMs and Bayesian networks to clarify links between EAP and cognition in large samples of schizophrenia patients and controls. The most robust findings across both networks and groups were that EAP and attention/vigilance measures were interconnected but conditionally independent from other cognitive domains used in this study. Even though MMN and P3a are widely presumed to reflect pre-attentive auditory information, this is the first study to provide evidence of their dependent relationships to performance-based measures of attention/vigilance while accounting for other cognitive processes. These findings support hierarchical information processing models of schizophrenia (Dondé, Silipo, Dias, & Javitt, 2019; Heinz et al., 2018; Rissling et al., 2012). The Bayesian networks offered additional insights into these relationships. In schizophrenia, MMN was a parent variable upon which P3a, DS-CPT, and CPT-IP depended. In addition, DS-CPT depended on P3a, forming a causal chain in schizophrenia (MMN → P3a → DS-CPT → CPT-IP). In controls, P3a also depended on MMN, but no variables depended on P3a. Hence, a slightly different causal chain emerged in controls (MMN → CPT-IP → DS-CPT). Differences in directional dependency may help explain attentional deficits in schizophrenia. As attention/vigilance are further down the estimated causal pathway, EAP measures could play a more influential role and contribute to more pronounced impairment in schizophrenia.

Using Bayesian networks, we were able to identify differences in the direction of influence of cognitive functions between the groups. In controls, the N-back was a key parent variable, and the VOLT was either a direct endpoint or the endpoint of a chain structure. In schizophrenia patients, the N-back was a collider and operated as a common effect, and the VOLT was a key parent variable that directly influenced multiple cognitive domains. However, removing the VOLT from the model altered each group's cognitive structure so that they were similar, suggesting that both groups may have the same default ‘baseline’ structure when this construct is not included in the model.

The strong effect size of the VOLT may help explain differences in Bayesian network structures and why the structures were similar after their omission. Because schizophrenia patients were more impaired on the VOLT, object memory may be viewed as a rate-limiting factor that affects cognitive performance across multiple domains. The importance of the VOLT in the structure of cognition is consistent with one top-down processing framework of visual perception (Adámek, Langová, & Horáček, 2022; Bar, 2003; Bar et al., 2006). According to this framework, object recognition is initiated in healthy individuals by top-down processes within the orbitofrontal cortex (OFC). Brain activation associated with successful object recognition develops and peaks in the OFC earlier than in more traditional visual processing regions, including the temporal cortex. As such, the OFC – and the prefrontal cortex in general – is thought to play a role in top-down predictions about the identity of objects. However, reduced neural activity in the OFC in schizophrenia patients may lead to disruptions in this top-down process. One interpretation of our results could be that controls first engage high-order cognitive functions, such as verbal and working memory, for successful task completion during the VOLT. In contrast, schizophrenia patients may not fully recruit high-order cognitive processes during the VOLT, contributing to a stronger reliance on bottom-up processing (Adámek et al., 2022; Javitt, 2009).

Our analytical approach adds novel insight into the relationship between EAP and cognition beyond what latent variable models have shown. In comparing our results with the previous SEM paper of the same dataset (Thomas et al., 2017), three advantages are worth highlighting. First, using SEM, EAP predicted a latent variable of cognitive domains; however, this variable excluded the attention/vigilance tasks. Thus, our analyses yield a clearer understanding of the relationship between EAP and cognition by demonstrating that attention/vigilance is the sole cognitive domain associated with EAP used in this study after accounting for the associations among all cognitive tasks. Second, SEM treated MMN and P3a as interchangeable parts of an underlying factor. However, as demonstrated by EEG, MMN temporally precedes P3a, but SEM does not appropriately capture this nuance. Through Bayesian networks, we were able to represent different components of EAP and clarify how neurophysiological phenomena are linked to higher-order cognitive phenomena. By using latent variables, prior analyses assumed that MMN and P3a had similar effects on cognition, potentially masking unique associations. Leveraging a complementary set of network analyses, we demonstrated that MMN and P3a have distinct effects on cognition. Third, by utilizing DAGs, we were able to identify different dependent structures that can inform statistical control and causal assumptions. For instance, in both groups, EAP is not associated with working memory given attention/vigilance and would be hypothesized to be conditionally independent (EAP ⊥ working memory | attention/vigilance).

One limitation was our reliance on cross-sectional data. Although there is growing emphasis on the need for longitudinal data in network models, EAP and cognition are relatively stable processes (McCleery & Nuechterlein, 2019; Pietrzak et al., 2009; Szöke et al., 2008). Nonetheless, future studies should examine the dynamic nature of cognition to uncover the appropriate timescale and temporal distance in which cognitive processes influence each other over time (Hopwood, Bleidorn, & Wright, 2022). Our results could be further advanced by examining longitudinal *idiographic* network models. Recent work indicates that cross-sectional network parameters from baseline partial correlation networks are predictive of within-subject networks over time (von Klipstein, Borsboom, & Arntz, 2021). Another limitation of the current study was that we did not experimentally control medication effects. We previously showed that the schizophrenia patients of COGS-2 have a high anticholinergic medication burden (ACB), which was associated with measures of EAP and cognition (Joshi et al., 2021). However, we chose not to adjust for ACB in the current analyses because it would not provide a valid comparison to the control networks. As such, we view our analyses and results as providing information on the fundamental structure of EAP and cognition. Similarly, our results are limited by the types of EAP and cognitive tasks included in the study. Namely, we did not include any behavioral measures of EAP, such as the Tone Matching Test. In addition, social cognitive tasks that measure auditory emotion recognition and measures of early visual processing, such as visual backward masking, could be relevant for understanding the causal pathways between EAP and cognition (McCleery et al., 2020). Lastly, our Bayesian network analyses may have violated the assumption of acyclicity, as certain domains could overlap and tap into mutually dependent cognitive processes (Gold et al., 2009).

## Conclusion

We used GGMs and Bayesian networks to examine undirected and directed connections among measures of EAP and cognition in schizophrenia. These results could help identify dependent structures that can guide statistical decisions, such as how accounting for certain variables may lead to biased effects. Secondary, our results could be used to help create experimental or longitudinal studies to help identify intervention targets and surrogate endpoints, such as MMN and the VOLT. Ultimately, these types of analyses may be informative for gaining mechanistic insights into the downstream contributions of EAP to more distal cognitive, clinical, and functional disability.

## Supporting information

Abplanalp et al. supplementary material Abplanalp et al. supplementary material

## Supplementary material

The supplementary material for this article can be found at https://doi.org/10.1017/S0033291724000023
