# Supplementary material for: Clarifying directional dependence among measures of early auditory processing and cognition in schizophrenia: leveraging Gaussian graphical models and Bayesian networks
Source: Psychol Med. 2024 Jan 30;54(9):1930–9. doi: 10.1017/S0033291724000023 (PMC11413352; doi:10.1017/S0033291724000023)
Supplement: Abplanalp et al. supplementary material [file S0033291724000023sup001.docx]

**Supplementary Material for Clarifying Directional Dependence Among Measures of Early Auditory Processing and Cognition in Schizophrenia: Leveraging Gaussian Graphical Models and Bayesian Networks**

**Supplementary Information 1**. Bivariate Correlation and Standard Partial Correlation Networks

**Supplementary Information 2**. GGM Accuracy and Stability

**Supplementary Information 3**. Bayesian Network Age Stratification Procedure

**Supplementary Information 4**. Estimating Regression Models Corresponding to Selected Dependent Structures

**Supplementary Figure 1**.Bivarite and Standard Partial Correlation Networks for Controls and Schizophrenia Patients.

**Supplementary Figure 2**. GGM Accuracy and Stability.

**Supplementary Figure 3**. Bayesian Networks for Controls and Schizophrenia Patients After Stratifying for Age.

**Supplementary Figure 4**. Illustration of the Dependent Structure LNS-F → LNS-R → CVLT for Controls and Schizophrenia Patients.

**Supplementary Figure 5**. Illustration of the Dependent Structure MMN → P3a → CPT-IP → DS-CPT for Controls and Schizophrenia Patients.

**Supplementary Information 1**. Bivariate Correlation and Standard Partial Correlation Networks

To illustrate how associations among measures of EAP and cognition can differ among correlation types, we estimated bivariate correlation and standard partial correlation (without regularization) networks in schizophrenia patients and controls. Both network types were once again estimated using the R package *bootnet* and plotted using *qgraph*. The networks are presented in Supplementary Figure 1. As we can see, both types of networks have a greater number of edges than the GGMs.

**Supplementary Information 2**. GGM Accuracy and Stability

We evaluated the accuracy of the GGM edge estimates in schizophrenia patients and controls by conducting the routine implemented in *bootnet* using nonparametric bootstrapping with 1,000 bootstrap samples (Epskamp et al., 2018). This procedure estimates the stability of GGM edges and provides confidence intervals for each edge. GGM accuracy and stability results are shown in Supplementary Figure 2. The confidence intervals are narrow for each edge and do not contain zero, suggesting highly stable edges for both networks.

**Supplementary Information 3**. Bayesian Network Age Stratification Procedure

To examine the potential impact of age on the Bayesian network results (Lee et al., 2020), we conducted a sensitivity analysis in which we stratified for age. We used the R package *MatchIt* to conduct the stratification procedures, with all code available in the OSF link provided in the main text. Nearest neighbor matching was used as the primary matching method. Briefly, nearest neighbor matching runs through the list of treated units and selects eligible control units to be paired with the treated unit—without regard to any optimization. In addition, we set a caliper of 0.1 (based on age propensity scores) to further ensure that the groups were matched for age. Both groups contained 497 participants after matching for age. The schizophrenia group had a mean age of 44.00, and the control group had a mean age of 43.28; this difference was not statistically significant *t*(991.29) = -0.98, *p* = 0.33, indicating appropriate matching.

After stratifying for age, we estimated Bayesian networks using the *bnlearn* R package (Supplementary Figure 3). We used the original network output from *bnlearn*. We chose not to modify the appearance because these networks were not being compared to any GGM equivalent. Minimal differences were observed between the unstratified and stratified networks. Like the unstratified networks, EAP variables were conditionally dependent on attention and conditionally independent of the other cognitive domains in both groups. In addition, the VOLT was still a key parent variable in schizophrenia patients (Panel B) and a descendant variable in controls (Panel A), and the N-back was a key parent variable in controls.

**Supplementary Information 4**. Estimating Regression Models Corresponding to Selected Dependent Structures

We conducted linear regression analyses to illustrate selected dependent structures estimated from the Bayesian networks and how they can be used to help inform statistical control. All R code for the analyses is available from the link in the main text. First, we estimated a model where the LNS-F is the dependent variable being predicted by the CVLT (Supplementary Figure 4) in both controls (Panel A) and schizophrenia patients (Panel B). This model corresponds to the Bayesian network structure LNS-F → LNS-R → CVLT, called a causal chain (known as a mediator). Supplementary Figure 4 illustrates why it is critical to account for the LNS-R when estimating a direct effect between the LNS-F and the CVLT. The LNS-F has an indirect effect on the CVLT *through* the LNS-R. As such, not accounting for the LNS-R will bias estimates, as we can see a robust difference in the Beta coefficients in both groups when accounting for its effects and when not accounting for its effects.

Supplementary Figure 5 illustrates a slightly different scenario from Supplementary Figure 4. Here, we estimate a direct effect between MMN (Dependent Variable) and the DS-CPT (Independent Variable). This model corresponds to the following dependent structure: MMN → P3a → CPT-IP → DS-CPT. The models for both controls (Panel A) and schizophrenia patients (Panel B) show the association between MMN and the DS-CPT while accounting for different combinations of variables, including P3a, the CPT-IP, both P3a and the CPT-IP, and neither P3a nor the CPT-IP. As a reminder, the Bayesian network for controls had a dependent structure of MMN → CPT-IP → DS-CPT, while the schizophrenia patients had MMN → CPT-IP → DS-CPT in addition to MMN → P3a → DS-CPT. Thus, we would only account for the CPT-IP to estimate a direct effect between MMN and the DS-CPT in controls. Meanwhile, in schizophrenia patients, we would account for the CPT-IP and P3a (both controls and schizophrenia patients’ correct level of control are illustrated by the red line). As shown in the Figure, these different levels of control result in similar estimates in control participants (Beta = -0.154) and schizophrenia patients (Beta = -0.151). However, accounting for the wrong combination of variables leads to biased estimates, as shown by the different colored lines. Supplementary Figures 4 and 5 are powerful demonstrations of how Bayesian networks can guide statistical control and ultimately help inform causal relationships.

**Supplementary Figure 1. Bivariate and Standard Partial Correlation Networks for Controls and Schizophrenia Patients.**

**
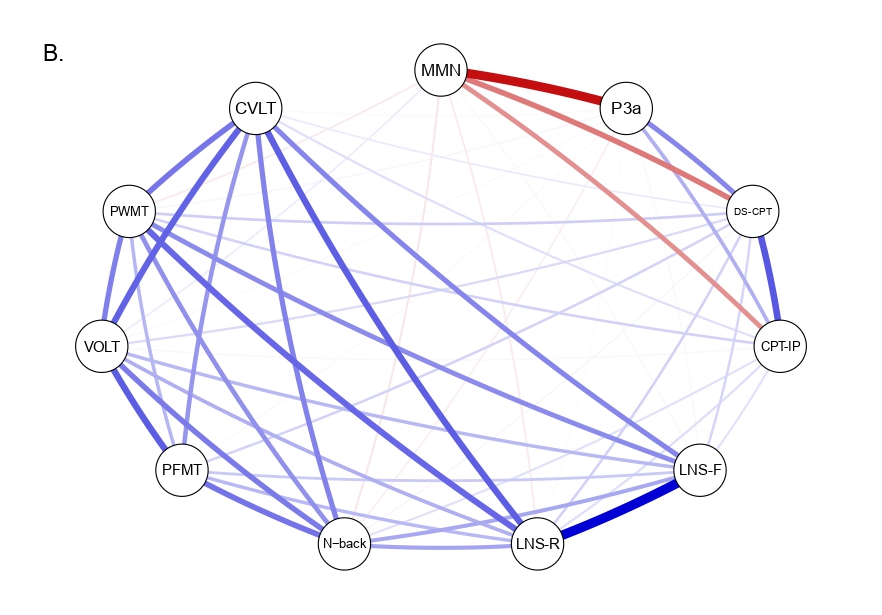

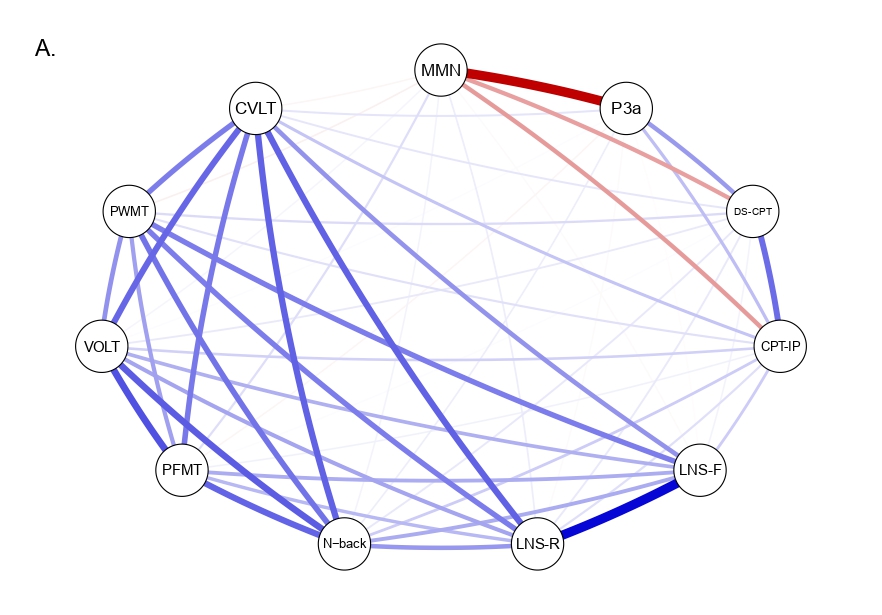
**

**
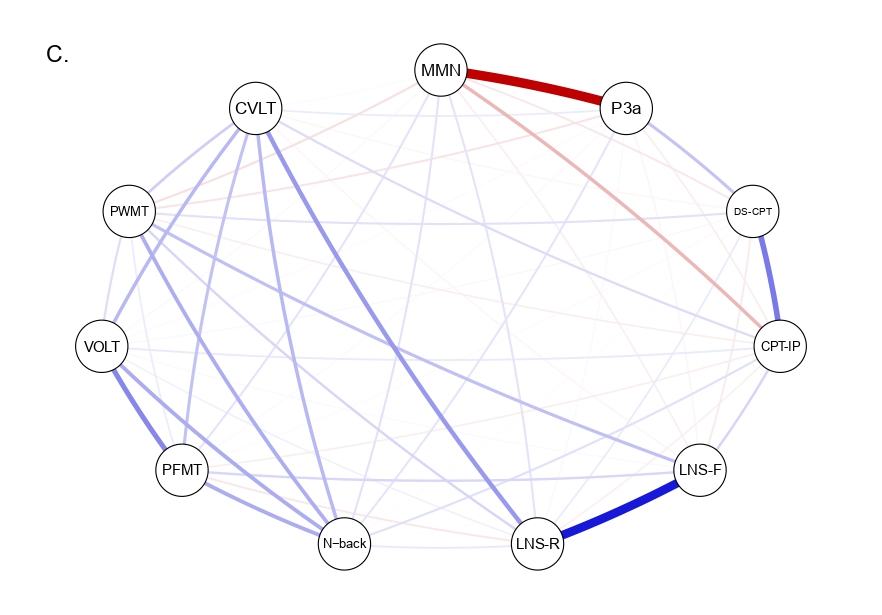

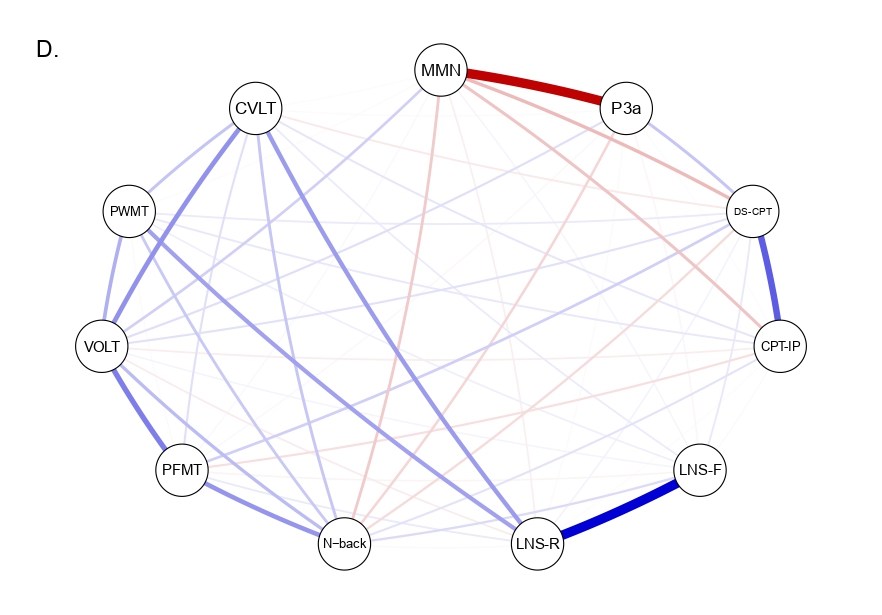
**

Note. Panel A = Bivariate correlation network for controls; Panel B = Bivariate correlation network for schizophrenia patients; Panel C = Standard partial correlation network for controls; Panel D = Standard partial correlation network for schizophrenia patients. MMN = Mismatch negativity, DS-CPT = Degraded stimulus continuous performance test, CPT-IP = Continuous performance test identical pairs, LNS-F = Letter-Number Span Task Forward, LNS-R = Letter-Number Span Task Reorder, PWMT = Penn Word Memory task, CVLT = California Verbal Learning Test, N-back = Letter N-back task, PFMT = Penn Face Memory task, VOLT = Visual Object Learning Test.

Note. GGM = Gaussian Graphical Model; Panel A = estimates for controls; Panel B = estimates for schizophrenia patients. The red line indicates the sample values, and the gray indicates the bootstrapped confidence intervals. Each horizontal line represents one edge of the network, ordered from the edge with the highest weight to the edge with the lowest weight. Edge weights (x-axis) sorted in increasing order (red line). The grey areas have 95% confidence intervals. y-axis-labels (i.e., edge-edge relationships) are omitted to limit unclear interpretation.


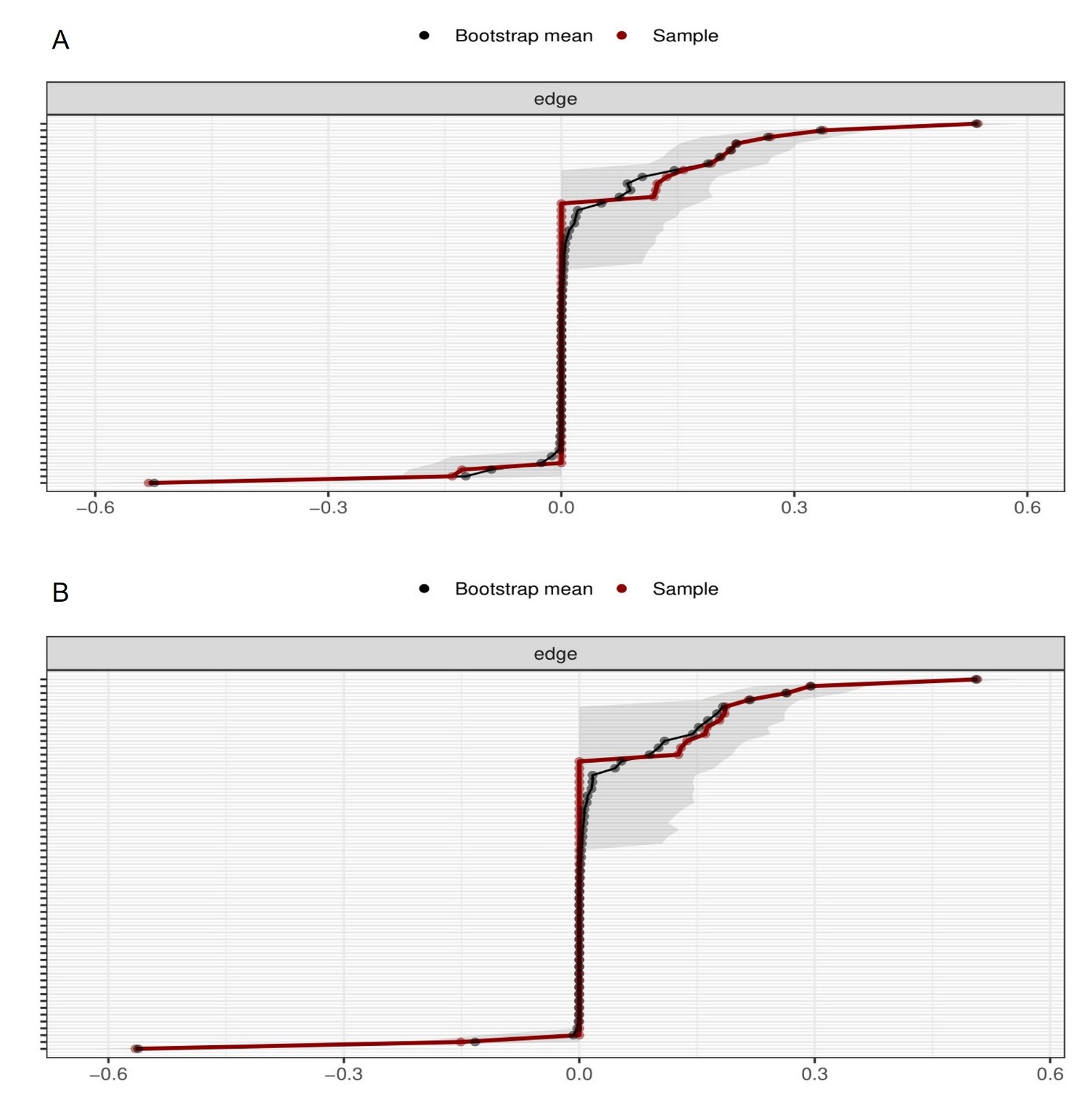


**Supplementary Figure 2. GGM Accuracy and Stability.**


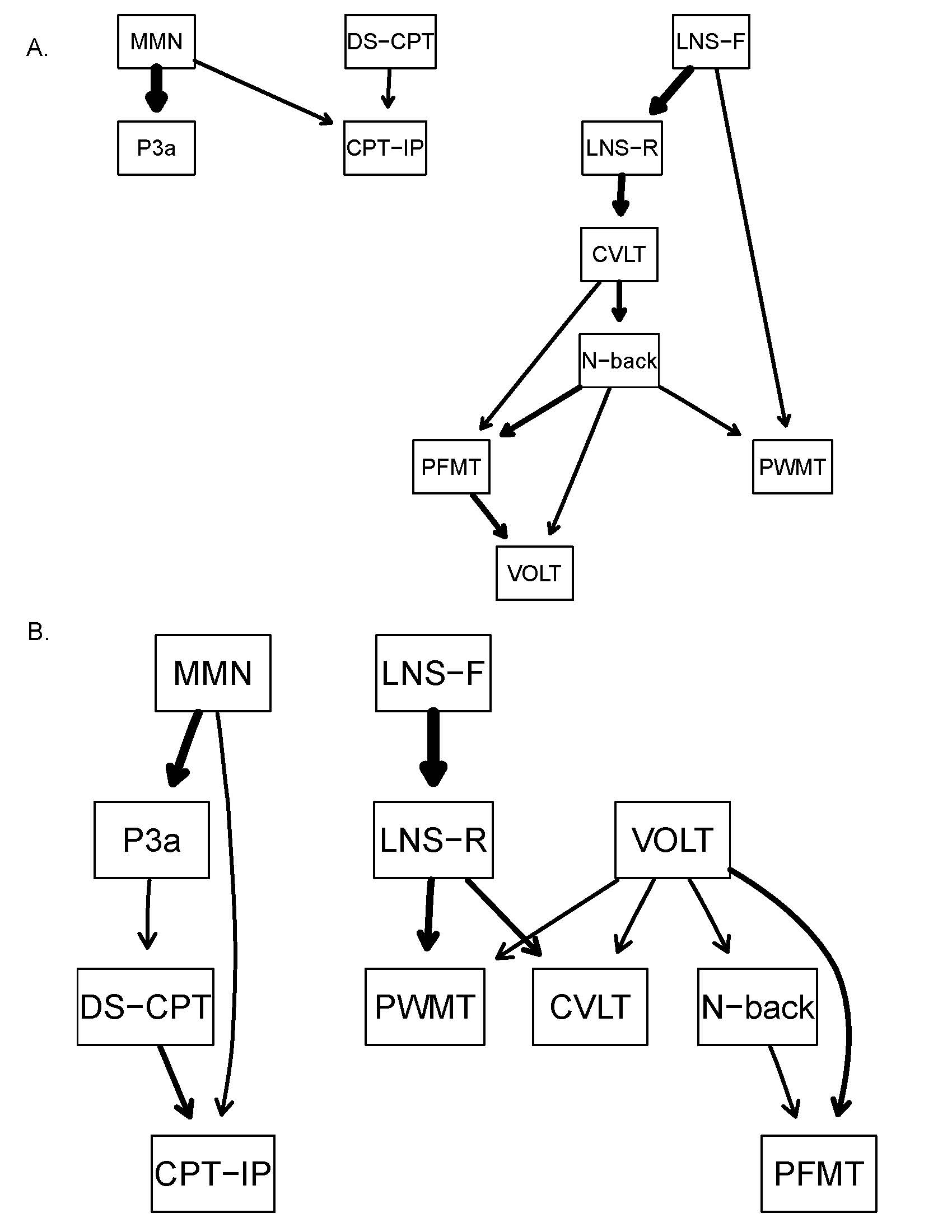


**Supplementary Figure 3. Bayesian Networks for Controls and Schizophrenia Patients After Stratifying for Age.**

Note. Panel A = Bayesian network for controls; Panel B = Bayesian network for schizophrenia patients. MMN = Mismatch negativity, DS-CPT = Degraded stimulus continuous performance test, CPT-IP = Continuous performance test identical pairs, LNS-F = Letter-Number Span Task Forward, LNS-R = Letter-Number Span Task Reorder, PWMT = Penn Word Memory task, CVLT = California Verbal Learning Test, N-back = Letter N-back task, PFMT = Penn Face Memory task, VOLT = Visual Object Learning Test.

Note. Panel A = Regression model for controls; Panel B = Regression model for schizophrenia patients; LNS-R = Letter-Number Span Task Reorder; LNS-F = Letter-Number Span Task Forward; CVLT = California Verbal Learning Test.
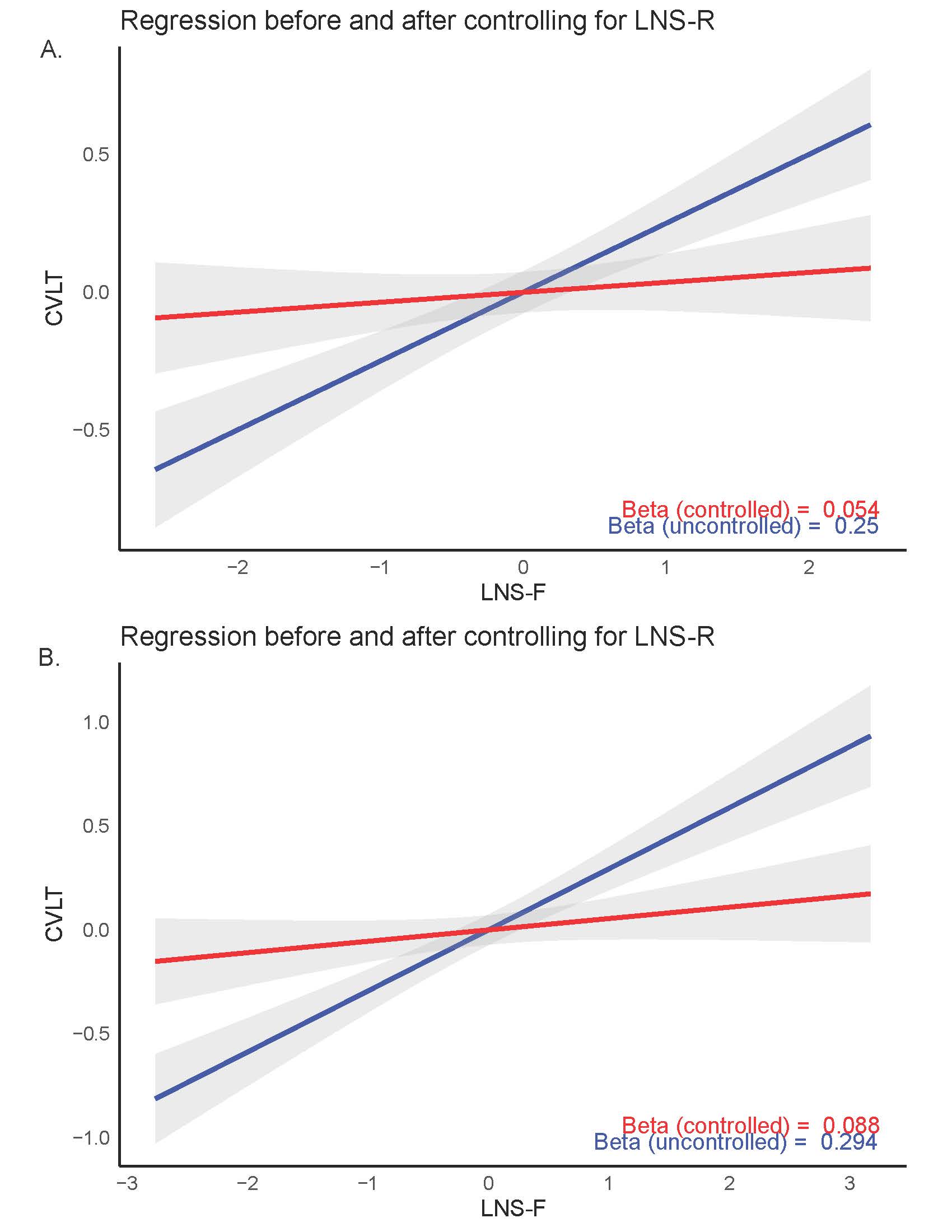


**Supplementary Figure 4. Illustration of the Dependent Structure LNS-F → LNS-R → CVLT for Controls and Schizophrenia Patients.**


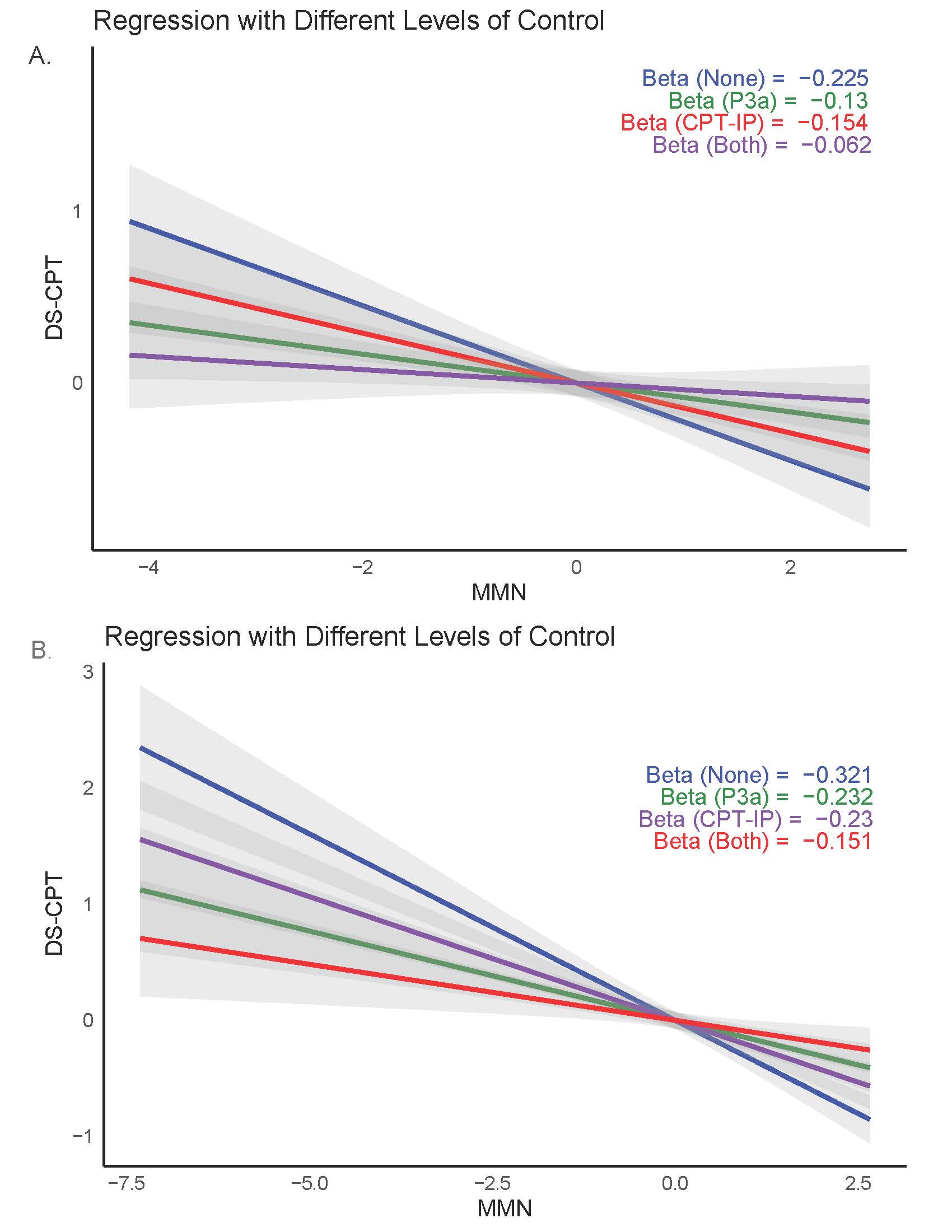
Note. Panel A = Regression model for controls; Panel B = Regression model for schizophrenia patients; MMN = Mismatch negativity, DS-CPT = Degraded stimulus continuous performance test, CPT-IP = Continuous performance test identical pairs.

**Supplementary Figure 5. Illustration of the Dependent Structure MMN → P3a → CPT-IP → DS-CPT for Controls and Schizophrenia Patients.**
